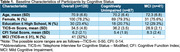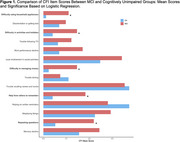# Evaluating the Relationship Between Cognitive Function Index and TICS‐m in Detecting Cognitive Impairment: Preliminary Results from the Remote‐CARE Study

**DOI:** 10.1002/alz70857_105059

**Published:** 2025-12-24

**Authors:** Babak Khorsand, Elham Ghanbarian, Hailey J. Andrews, Hannah Bodek, Jack D. Cameron, Chloe Moffitt, Robert Lavin, Richard B. Lipton, Laura A. Rabin, Nelson A. Roque, Nicole Sergeyev, Ali Ezzati

**Affiliations:** ^1^ University of California, Irvine, Irvine, CA, USA; ^2^ The Pennsylvania State University, University Park, PA, USA; ^3^ Albert Einstein College of Medicine, Bronx, NY, USA; ^4^ Brooklyn College of the City University of New York, Brooklyn, NY, USA

## Abstract

**Background:**

The Cognitive Function Index (CFI) and the Modified Telephone Interview for Cognitive Status (TICS‐m) are widely used tools for cognitive screening in older adults. TICS‐m is an examiner‐administered, objective test, while the CFI is a self‐reported, subjective questionnaire. Although subjective cognitive concerns are common among older adults and may portend progression to dementia, the cross‐sectional relationship between subjective and objective cognition is inconsistent. This study examines the association between CFI and TICS‐m to evaluate the utility of self‐reported cognitive concerns in identifying cognitive impairment in older adults.

**Method:**

The Remote Cognitive Aging and Alzheimer's Disease REsearch (*R*‐CARE) study is an ongoing initiative aimed at validating a remote cognitive assessment toolbox for diverse, dementia‐free older adults. As part of the screening for this cohort, 1496 individuals identified through the EHR system were contacted, 158 agreed to participate, and 128 participants completed both CFI and TICS‐m assessments. Mild cognitive impairment (MCI) was defined as an education adjusted TICS‐m cut‐score (Knopman et al., 2010). Linear regression assessed the relationship between CFI and TICS‐m scores, while logistic regression evaluated the ability of CFI items to classify cognitively unimpaired (CU) and MCI participants, defined by the TICS‐m, adjusting for demographic covariates.

**Results:**

Participants had a mean age of 70.9±6.6 years, and 32% were categorized as MCI based on TICS‐m scores. Higher CFI total scores were significantly associated with lower TICS‐m scores in linear regression models (ß=‐0.47±0.18, *p* = 0.009). Logistic regression models identified five CFI items significantly associated with MCI classification after adjusting for age, sex, education, and race: repeating questions (OR=1.88, *p* = 0.01), needing help to remember (OR=1.63, *p* = 0.04), difficulty managing money (OR=1.77, *p* = 0.03), difficulty in activities (OR=1.58, *p* = 0.04), and difficulty using household appliances (OR=2.48, *p* = 0.03).

**Conclusion:**

The Cognitive Function Index (CFI) showed a significant association with TICS‐m scores, highlighting its potential as a complementary tool for identifying cognitive impairment. Specific self‐reported items, such as repeating questions and difficulties with activities of daily living, were predictive of MCI. These findings suggest that CFI, with its brevity and ease of administration, may prove valuable for remote cognitive screening and to enhance early detection efforts in diverse populations.